# A case report of Hodgkin lymphoma in a patient treated with ustekinumab for psoriasis

**DOI:** 10.1097/MD.0000000000020048

**Published:** 2020-05-22

**Authors:** Emmanouil Charakopoulos, Ioannis Spyrou, Nora-Athina Viniou, Nefeli Giannakopoulou, Sevastianos Hatzidavid, Panagiotis Theodorou Diamantopoulos

**Affiliations:** Hematology Unit, First Department of Internal Medicine, Laikon General Hospital, National and Kapodistrian University of Athens, Athens, Greece.

**Keywords:** Hodgkin lymphoma, psoriasis, ustekinumab

## Abstract

**Rationale:**

Ustekinumab is a biological agent that inhibits interleukin 12 and 23 and has been approved for the treatment of moderate and severe plaque psoriasis. There have been case reports that raise concerns about its oncogenic potential. We are the first authors to report a case of Hodgkin lymphoma in a psoriatic patient receiving ustekinumab.

**Patient concerns:**

A 22-year-old asymptomatic female patient presented to our department to investigate an enlarged cervical lymph node. Her past history was unremarkable, except for psoriasis since age 13. Two months before presentation the decision to administer Ustekinumab was taken and the patient had already received 3 doses.

**Diagnoses:**

During workup a Stage IV Hodgkin lymphoma was discovered.

**Interventions:**

Ustekinumab administration was discontinued. The patient received treatment with the ABVD regimen.

**Outcomes:**

The patient's disease was refractory to the above-mentioned treatment. Therefore, a more aggressive regimen (BEACOPP escalated) was administered.

**Lessons:**

Growing postmarketing surveillance data and case reports indicate that further research is warranted in order to elucidate a potential association between Ustekinumab and malignancy.

## 1 Introduction

Ustekinumab (Stelara) is a new biologic agent, which is primarily used in the treatment of moderate to severe plaque psoriasis.^[1]^ It has been proven to be effective and safe in the vast majority of studies,^[2–11]^ but there are case reports of malignancies that have been associated with ustekinumab administration.^[12–16]^ We present a case of a young female patient who was diagnosed with Hodgkin lymphoma (HL) 2 months after treatment initiation with ustekinumab for severe psoriasis. In the literature, we detected similar reports that correlated ustekinumab with hematologic malignancies, more specifically multiple myeloma,^[16]^ cutaneous T-cell lymphoma,^[13]^ and gastric mucosa-associated lymphoid tissue lymphoma.^[15]^

## 2 Case presentation

A 22-year-old woman with chronic plaque psoriasis since the age of 13, and an otherwise unremarkable past medical history, was diagnosed with HL after being evaluated for a painless enlarged supraclavicular lymph node. There was no account of B-symptoms. The family history had also been negative for malignancy. The patient is a former smoker (3.5 pack-years exposure) and drinks socially. No allergies were reported.

In the initial workup that followed the clinical examination, white blood cell and platelet counts were slightly elevated, with 11 × 10^9^ cells/L and 480 × 10^9^ platelets/L respectively; the remaining values of the complete blood count and routine biochemical assays were within normal range. An excisional biopsy was performed and the pathology examination established the diagnosis of HL, subtype nodular sclerosis. For staging purposes, a bone marrow biopsy and imaging studies were performed. A positron emission tomography/computed tomography demonstrated multiple foci of increased bone marrow uptake, suspicious of malignant infiltration. The bone marrow biopsy reported focal reactive changes with dysplastic features, with no confirmed infiltration by HL. Imaging revealed an extensive lymph node involvement (cervical, axillary, mediastinal, pulmonary hilar bilaterally, splenic hilar, left inguinal, paraaortic, iliac, and mesenteric nodes). Finally, a solitary slightly hypermetabolic nodular lesion was detected in the left lung parenchyma, which was; however, deemed unlikely to stem from lymphoma cells. Thus, the disease was determined to be stage IV with the Cotswold modification of the Ann Arbor staging system.

The patient was treated with ABVD chemotherapy (doxorubicine, bleomycin, vinblastine, dacarbazine) according to the common clinical practice in our institution, in an outpatient setting; an interim positron emission tomography/computed tomography was performed after the second cycle. Abnormally hypermetabolic lymph nodes were detected in the cervical, thoracic and abdominal region; these included paratracheal and anterior mediastinal lymph nodes, as well as 2 foci in the splenic parenchyma among other sites (4 points in the Deauville scoring scale indicating active residual disease).^[17]^ The drug regimen was modified to BEACOPP escalated (bleomycin, etoposide, doxorubicine, cyclophosphamide, vincristine, procarbazine, prednisone).

Regarding the course of psoriasis, it is worth mentioning that the initial psoriatic plaque was located on the scalp; therefore, a topical corticosteroid was prescribed. However, patient adherence to treatment was poor and psoriatic lesions were generalized along the extremities and trunk over a 2-year period. The treating dermatologist opted for a combination of topical treatments which led to partial regression of the psoriatic plaques, over the next 6 years. Ultimately, 2 months before the diagnosis of the HL, treatment with ustekinumab was started. A dose of 90 mg (according to the patient's weight) was administrated subcutaneously at that point^[1]^; another dose followed 1 month later and the last one a few days before the diagnosis of HL. The treatment led to complete regression of the cutaneous lesions, even from the first injection of ustekinumab. Because of the diagnosis of HL, ustekinumab was discontinued. Lesions then gradually recurred on the scalp of the patient in the next months and are now under partial control with the application of budesonide, mometasone, salicylic acid, and an emollient cream.

## 3 Discussion

Ustekinumab is a new monoclonal antibody that has been approved by the U.S. Food and Drug Administration (FDA) for the treatment of moderate to severe plaque psoriasis since 2009.^[18]^ Its mechanism of action is unique: it binds to and inhibits the common subunit (p40) of Interleukin (IL) 12 and 23; therefore, reducing inflammatory response.^[19]^ In psoriasis, IL-12 and IL-23 induce inflammation through the activation of T-helper 1 and T-helper 17 cells respectively.^[20,21]^ Other indications of ustekinumab are Crohn disease, psoriatic arthritis, and relapsing multiple sclerosis.^[19]^

Despite the fact that the simultaneous blockade of the IL-12 and IL-23 pathway is oncogenic in experimental animal models,^[22,23]^ the safety and efficacy of ustekinumab in the treatment of psoriasis has been well demonstrated in 3 phase III clinical trials: PHOENIX 1,^[8]^ PHOENIX 2,^[24]^ and ACCEPT.^[25]^ The most common side effect was grade 2 upper airway tract infections (according to Common Terminology Criteria for Adverse Events v4.0).^[26]^ Although there were reports of malignancies, there was no observable statistically significant difference compared to the placebo groups.^[19]^ However, the results of those trials cannot guarantee the long term safety of ustekinumab. An instructive example is efalizumab, which had been proven to be effective and safe in the treatment of psoriasis by inhibiting the leukocyte function-associated antigen 1^[27]^ before its withdrawal from the market in 2009, because of its potential to cause progressive multifocal leukoencephalopathy.^[28]^

Several authors in the literature report cases of malignancies in patients treated with ustekinumab.^[12–16]^ These cases are displayed concisely in Table 1.

**Table 1:**
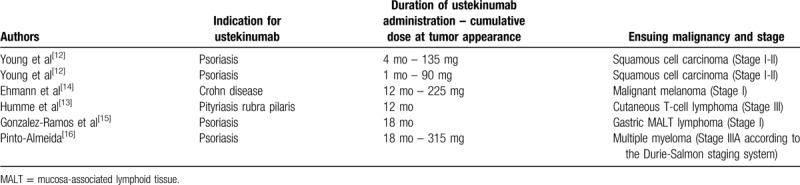
Case reports with emergence of malignancies in patients under treatment with ustekinumab.

Nevertheless, before attributing the patient's HL to ustekinumab, the inherent risk of malignancy in psoriatic patients should also be taken into consideration. Several studies have shown an overall increased cancer risk in patients with psoriasis, even before the use of biologics in its treatment. Lymphoma, nonmelanoma skin cancer, and cancers associated with smoking and alcohol consumption are the most consistently reported malignancies.^[29–42]^ The risk of developing cancer is proportionate to disease severity^[30,32]^ and might be influenced by gender and age. Young males seem to represent the group at the highest risk.^[42]^

The increased risk of lymphoma in psoriasis is consistent with the pathophysiology of this chronic inflammatory skin disease, because it is characterized by a diffuse cytokine-induced immunological activation.^[32]^ Psoriasis has been shown to increase the risk of all lymphoma types,^[30,31,36,39,43,44]^ especially of HL according to some studies.^[30,39]^ Gu et al conclude that psoriasis might also increase the risk of lymphoma in pediatric patients.^[45]^ Because of the above evidence, we cannot exclude the possibility that our patient's HL is spontaneous or that it is associated with psoriasis and not ustekinumab, but the emergence of such an advanced lymphoma after the initiation of treatment makes these alternative hypotheses less probable.

On the other hand, the relatively short treatment duration cannot fully explain the development of such an advanced tumor. Experiments in primates showed that the administration of an extremely high dose of IL-12/IL-23 inhibitor did not cause cancer for a minimum of 6 months.^[46]^ Therefore, it could be hypothesized that ustekinumab led to an exacerbation of an already existing subclinical malignancy in our patient. This assumption is in agreement with the supposition of Young and Czarnecki, who claim that the immunosuppressive effect of ustekinumab might cause a subclinical malignancy to become apparent.^[12]^ As mentioned in the relative prescription information of Stelara, little is known about the effects of ustekinumab in patients with a known cancer, because most patients with a history of malignancy were excluded from PHOENIX 1, PHOENIX 2, and ACCEPT trials as ineligible.^[8,24,25,47]^ More studies are needed to determine if a known history of malignancy represents a contraindication to the initiation of treatment with ustekinumab.

In November 2017, Florek et al, as part of the postmarket surveillance between 2001 and 2016, reported a detected safety signal in the voluntary FDA Adverse Event Reporting System concerning B-cell lymphoma, lung, thyroid, and ovarian cancer among others; similar reports arose also in the EudraVigilance database. Despite the possible presence of reporting bias, and the lack of details that could augment our effort to establish potential causality (dose, duration of exposure, timing of manifestation of malignancy) and elucidate possible confounding factors and after taking into account the fact that a causal relationship need not be determined for the FDA in these reports, it should be emphasized that postmarket surveillance garners real world data that may elude randomized control trials. Therefore, it is clear that further long term studies are warranted to investigate the possible link between ustekinumab and the risk of malignancy.^[48]^

## 4 Conclusions

In conclusion, ustekinumab might increase the risk of certain malignancies. This is in accordance with various case reports and postmarket surveillance data. To our knowledge, this is the first recorded case of a HL in a patient receiving ustekinumab. More studies are needed to determine a potential link between ustekinumab and cancer.

## Author contributions

**Conceptualization:** Nora-Athina Viniou, Nefeli Giannakopoulou, Sevastianos Hatzidavid, Panagiotis T. Diamantopoulos.

**Formal analysis:** Emmanouil Charakopoulos.

**Methodology:** Emmanouil Charakopoulos, Ioannis Spyrou, Panagiotis T. Diamantopoulos.

**Project administration:** Ioannis Spyrou, Nefeli Giannakopoulou, Sevastianos Hatzidavid.

**Resources:** Emmanouil Charakopoulos.

**Software:** Emmanouil Charakopoulos.

**Supervision:** Nora-Athina Viniou, Panagiotis T. Diamantopoulos.

**Validation:** Panagiotis T. Diamantopoulos.

**Writing – original draft:** Emmanouil Charakopoulos, Ioannis Spyrou.

**Writing – review and editing:** Emmanouil Charakopoulos, Ioannis Spyrou.
